# Announcement: 5th European Biological Inorganic Chemistry Conference (Eurobic-5)

**DOI:** 10.1155/MBD.1999.197

**Published:** 1999


					EuR
eO.ne',C-5
5th Europ Bological
Inorganic Chemistry Conference
Toulouse, France, July 17-20, 2000
INTERNATIONAL ORGANIZING COMMITEE
S. FORSEN, (Lund, Denmark)
F. GE)NZALEZ-VILCHEZ (Seville, Spain)
B. MEUNIER (Toulouse, France)
M. A. DE LA ROSA (Seville, Spain)
R. N. F. THE)RNELEY (Norwich, United Kingdom)
J. ULSTRUP (Lyngby, Denmark)
R. WEISS (Strasbourg, France)
NATIONAL ORGANIZING COMMITTEE
B. MEUNIER, Chairman (Toulouse)
R. WEISS, Co-Chairman (Strasbourg)
J. BERNADE)U, Treasurer (Toulouse)
D. NEIBECKER, Vice-Treasurer (Toulouse)
G. BALAVOINE (Bruxelles)
P. BERTRAND (Marseille)
J.-J. BONNET (Toulouse)
M. BRUSCHI (Marseille)
J.-C. CHE)TTARD (Paris)
M. COMTAT (Toulouse)
M. FONTECAVE (Grenoble)
J.-J. GIRERD ()rsay)
R. GUILARD (Dijon)
J.-P. LAUNAY (Toulouse)
D. MANSUY (Paris)
I. MORGENSTERN (Orsay)
J.-P. SAUVAGE (Strasbourg)
G. SIMONNEAUX (Rennes)
J.-B. VERLHAC (Bordeaux)
LOCAL ORGANIZING COMITTEE
A. BACEIREDO
J. BERNADOU
A.-M. CAMINADE
R. CHAUVIN
J.-P. COSTES
C. HEMMERT
G. LAVIGNE
N. LUGAN
E. MANOURY
B. MEUNIER
G. PRATVIEL
A. ROBERT
197
SCIENTIFIC PROGRAMME
EUROBIC-5 will include the following themes:
Biocatalysis with metalloenzymes and related models
Electron transfers in biological systems
Metalloproteins
Metals in medicine
Metals in the environment
Nucleic acids and metal complexes
Physical methods in bioinorganic chemistry
The symposium will hold 10 plenary lectures, 26 section lectures in 2 parallel sessions, 8 oral
presentations of selected posters, and 2 poster sessions.
PLENARY LECTURERS
The following prominent scientists have already agreed to present plenary lectures
I. BERTINI (Florence, Italy): Topic: Progress in NMR of metalloproteins.
J. T. GROVES (Princeton, USA): Topic: Peroxynitrite and metal complexes
B. KREBS (Menster, Germany): Topic: Phosphatase.
J.-M. LEHN (Strasbourg, France):Topic: Supramolecular chemistry.
D. MANSUY (Paris, France): Topic: NO synthase.
J. J. G. MOURA (Monte Caparica, Portugal): Topic: ATP sulfurylase (Co-Zn protein).
P. NORDLUND (Stockholm, Sweden): Topic: X-ray of RNR.
J. REEDIJK (Leiden, The Netherlands): Topic: Platinum chemistry and DNA.
D. S. SIGMAN (Los Angeles, USA): Topic: DNA cleavage.
R. N. F. THORNELEY (Norwich, United Kingdom):Topic: Nitrogen cycle.
GENERAL INFORMATIONS
The region "Midi-Pyrnes", located in Southwest France, offers a sumptuous historical heritage of
ancient monuments, castles, romanesque churches, villages (Cordes) and towns (Albi, Cahors,
Carcassonne). Its capital, the ancient city of Toulouse has become home to high technologies
linked to aircraft and space industries, electronics, and pharmaceuticals. The quality life-style,
famous gastronomy justifying the French paradox (foie-gras, magret, cassoulet, plus local wines),
and singing "southern" accent make Toulouse and its region pleasant places to live and to visit.
Toulouse can be reached by air (Toulouse-Blagnac International Airport) or TGV from the main
European cities. Accommodation will be available in comfortable hotels located downtown, within
walking distance of both the conference site and the historical city centre.
The symposium will be held in the "Centre de Congrs Pierre Baudis" located downtown Toulouse
SOCIAL PROGRAMME
An attractive social programme, including welcoming reception, banquet and concert will be
proposed to all participants. A special programme will be available for accompanying persons.
CORRESPONDENCE ADDRESS
EUROBIC-5
Laboratoire de Chimie de Coordination du CNRS
205, route de Narbonne
F-31077 Toulouse Cedex 04, France
Phone: + 33 5 61 33 31 00
Fax: + 33 5 61 55 30 03
E-mail: eurobic5 @ Icc-toulouse.fr
198

				

